# Engineered RNase P Ribozymes Effectively Inhibit Human Cytomegalovirus Gene Expression and Replication

**DOI:** 10.3390/v6062376

**Published:** 2014-06-13

**Authors:** Zhu Yang, Gia-Phong Vu, Hua Qian, Yuan-Chuan Chen, Yu Wang, Michael Reeves, Ke Zen, Fenyong Liu

**Affiliations:** 1 Institute of Virology, School of Life Sciences, Nanjing University, Nanjing, Jiangsu 210093, China; E-Mails: nooney1986@163.com (Z.Y.); kzen@nju.edu.cn (K.Z.); 2 Taizhou Institute of Virology, Taizhou, Jiangsu 225300, China; E-Mail: sin_angel@foxmail.com; 3 Jiangsu Affynigen Biotechnologies, Inc., Taizhou, Jiangsu 225300, China; 4 Program in Comparative Biochemistry, University of California, Berkeley, CA 94720, USA; E-Mails: giaphongvu@berkeley.edu (G.-P.V.); yuchuan1022@berkeley.edu (Y.-C.C.); 5 Department of Gynecology, People’s Hospital of Taizhou, Taizhou, Jiangsu 225300, China; E-Mail: huaqian1965@hotmail.com; 6 School of Public Health, University of California, Berkeley, CA 94720, USA; E-Mail: mreeves@berkeley.edu

**Keywords:** ribozyme, RNase P, gene targeting, cytomegalovirus

## Abstract

RNase P ribozyme can be engineered to be a sequence-specific gene-targeting agent with promising application in both basic research and clinical settings. By using an in vitro selection system, we have previously generated RNase P ribozyme variants that have better catalytic activity in cleaving an mRNA sequence than the wild type ribozyme. In this study, one of the variants was used to target the mRNA encoding human cytomegalovirus (HCMV) essential transcription factor immediate-early protein 2 (IE2). The variant was able to cleave IE2 mRNA *in vitro* 50-fold better than the wild type ribozyme. A reduction of about 98% in IE2 expression and a reduction of 3500-fold in viral production was observed in HCMV-infected cells expressing the variant compared to a 75% reduction in IE2 expression and a 100-fold reduction in viral production in cells expressing the ribozyme derived from the wild type sequence. These results suggest that ribozyme variants that are selected to be highly active *in vitro* are also more effective in inhibiting the expression of their targets in cultured cells. Our study demonstrates that RNase P ribozyme variants are efficient in reducing HCMV gene expression and growth and are potentially useful for anti-viral therapeutic application.

## 1. Introduction

Human cytomegalovirus (HCMV) is a common herpesvirus and usually affects individuals with compromised immune system [1]. This virus causes birth defects including mental problems [2]. Furthermore, HCMV can cause debilitating symptoms in AIDS patients, such as blindness and gastrointestinal diseases [3,4]. New therapeutic approaches are needed to combat and control this important opportunistic pathogen.

Gene targeting approaches using nucleic acid-based molecules to target specific mRNA sequences of choice represent promising therapeutic strategies [5,6]. One example is small interfering RNAs (siRNAs), which were used to target several human viruses effectively [5,7,8]. Ribozymes can also inactivate viral mRNA sequences and diminish viral infection in human cells [9,10,11,12].

**Figure 1:**
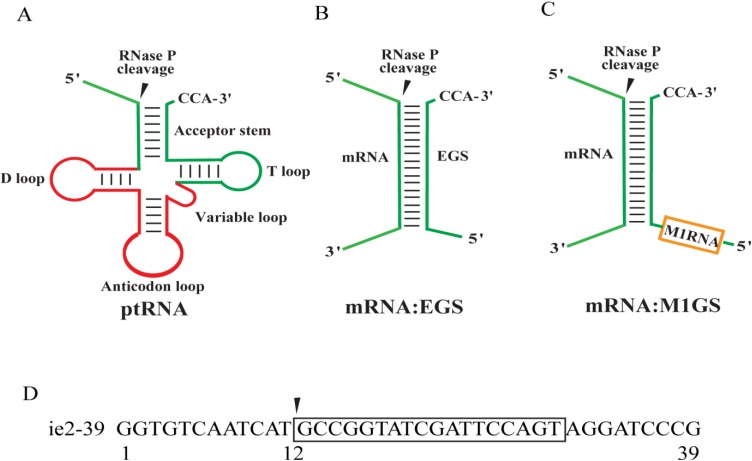
Schematic presentation of RNase P/M1GS ribozyme’s substrates. (**A**–**C**) pre-tRNA (ptRNA), a natural substrate (**A**); EGS:mRNA complex (**B**); and M1GS binding to its substrate (**C**). Filled arrows denote the cleavage sites. (**D**) Substrate ie2-39 with boxed region representing the complementary sequence to the guide sequence of M1GS.

RNase P functions in cells to process tRNA precursors (ptRNA) into mature tRNAs [13,14,15]. In *Escherichia coli*, RNase P forms a holoenzyme containing a protein (C5 protein) and a catalytically active RNA (M1 RNA) [16]. Previous studies have shown that the enzyme recognizes its substrate through the tertiary structure and not the primary sequence. (Figure 1A) [13,14,17]. Any mRNA substrate can be targeted and hydrolyzed by M1 ribozyme through the binding of a complementary external guide sequence (EGS) to the substrate to resemble the structure of the tRNA module that includes the 5’ leader sequence, the acceptor stem, the T-stem, and the 3' CCA sequence (Figure 1) [18,19]. A ribozyme targeting any mRNA sequence of choice, M1GS RNA, can be designed by linking a guide sequence (GS) to the 3' terminus of M1 RNA (Figure 1C) [20,21]. The guide sequence can basepair with an mRNA target and thus, allows M1GS to bind to the mRNA. Previous studies had designed M1GS ribozymes to target cellular genes and the essential genes of herpes simplex virus 1 (HSV-1) and HCMV [22,23,24]. The ribozymes were effective in reducing HSV-1 and HCMV growth 1000-fold and 150-fold, respectively [22,23].

Compared to other gene-targeting approaches such as RNAi, the M1GS ribozyme has several advantages. Ribozymes have not been shown to saturate the cellular machinery required for their processing while high levels of siRNAs can overwhelm the RNAi machinery that contains various cellular factors and may affect the normal cellular functions of these factors [5,25,26,27,28]. Furthermore, by interacting with specific cellular proteins including the RNase P protein subunits [14,29], RNase P ribozymes may improve their intracellular stability and catalytic activities in cells. RNase P ribozyme represents a unique and promising class of gene-targeting agents for therapeutic application [30].

Increasing the catalytic efficiency of the RNase P ribozyme is essential for M1GS technology to be used as an effective gene-targeting approach. Previously, we have generated RNase P ribozyme variants through an *in vitro* selection system that have better catalytic activity in cleaving an mRNA sequence than the wild type M1 RNA ribozyme [31]. For the study, a highly active ribozyme variant was designed to hydrolyze the exon 5 region of the mRNA encoding HCMV immediate-early 2 (IE2) protein. IE2 encodes a HCMV major transcription regulatory factor and its expression is needed for the expression of viral early (β) and late (γ) genes [1,32,33,34]. Thus, IE2 protein expression is essential for HCMV replication in cultured cells. Inhibition of IE2 expression would result in shutting down gene expression and replication of HCMV. We investigated the activity of the constructed ribozyme in generating cleavage of IE2 mRNA sequence *in vitro* and in shutting down HCMV infection in cells. Our data showed that the ribozyme variant cleaved the target IE2 mRNA sequence at least 50-fold more efficiently than the wild type ribozyme. More importantly, greater reduction in IE2 expression and HCMV growth was observed in cells expressing the variant. Our results suggest that RNase P ribozyme variants can be used as promising gene-targeting agents for anti-CMV applications.

## 2. Results

### 2.1. Targeting and Cleavage Activities of the M1GS RNAs in Vitro

Since most mRNA species inside cells form complex secondary structures and are associated with proteins, a targeting region must be accessible for ribozyme binding and catalytic cleavage of the target mRNA. We used dimethyl sulphate (DMS) [21,35] to map the accessible regions of IE2 mRNA *in vivo* [36]. To map these regions, we infected human U373MG cells with HCMV and then incubated these cells with culture media that contained DMS [36]. It is expected that DMS would enter the cells and modify the nucleotides of the accessible mRNA regions. We then isolated the total mRNAs. Primer extension assays using reverse transcriptase were used to map the IE2 mRNA regions that were modified by DMS [36]. A position, 30 nucleotides downstream from the 5' terminus of exon 5, was selected as the targeting site. The chosen targeting region (designated as IE2 RNA) was extensively modified by DMS and was predicted to be accessible for M1GS binding and cleavage (data not shown).

We had previously carried out an *in vitro* selection procedure and isolated M1GS RNA variants that have higher catalytic activity in cleaving HSV-1 thymidine kinase (TK) mRNA than the wild type M1 RNA [31]. However, little research has been done to examine whether these variants are also effective in targeting HCMV mRNAs to shut down viral infection. To investigate these issues, we chose variant 661 (designated as V661) for the study because this selected variant is highly active in cleaving target RNAs *in vitro* (e.g., IE2 and TK and mRNA) (Table 1) [31]. This variant contains two point mutations (*i.e.*, A_94_ → G_94_ and G_194_ → C_194_) [31]. Little is known about the contribution of these nucleotides to the activity of M1GS RNAs in cleaving an mRNA substrate. The effect of the point mutations on the activity of RNase P ribozymes has not been studied.

**Table 1:** Overall cleavage rate [(k_cat_/K_m_)^s^] and binding affinity (K_d_) of RNase P ribozymes with substrate ie2-39.

Enzyme	(k_cat_/K_m_)^s^ (µM^−1^·min^−1^)	K_d_ (nM)
M1-IE2	0.20 ± 0.05	0.34 ± 0.06
V661-IE2	10.5 ± 0.5	0.30 ± 0.05
M1-IE2-C	<5 × 10^−6^	0.33 ± 0.07
V661-IE2-C	<5 × 10^−6^	0.32 ± 0.07
M1-TK	<5 × 10^−6^	ND

The values shown are the average derived from triplicate experiments. *p < 0.01.* “ND”, not determined.

To construct IE2 mRNA-cleaving ribozymes from the variant and wild type M1 sequence, V661-IE2 and M1-IE2, the 3' termini of V661 and M1 RNA were covalently linked with an 18 nucleotides guide sequence that binds to the IE2 mRNA sequence, respectively. We included two control RNase P ribozymes, M1-IE2-C and V661-IE2-C. M1-IE2-C was generated from C102 RNA, an M1 mutant with mutations (A_347_C_348_ → C_347_U_348_, C_353_C_354_C_355_G_356_ → G_353_G_354_A_355_U_356_) at the catalytic P4 domain that rendered it inactive in cleaving a pre-tRNA [37]. V661-IE2-C was generated from V661-IE2 and contained the mutations found in C102. While sharing identical guide sequence with M1-IE2 and V661-IE2, both M1-IE2-C and V661-IE2-C were not expected to be functional because of the P4 mutations.

We measured the catalytic activity (k_cat_/K_m_)^s^ with kinetic analyses for these ribozymes in cleaving substrate ie2-39 of the 39 nucleotide long IE2 mRNA target sequence (Figure 1D). These results, shown in Table 1, indicate that V661-IE2 is about 50-fold more active than M1-IE2 in cleaving substrate ie2-39 (*p* < 0.01). As expected, V661-IE2-C and M1-IE2-C were at least 10^4^-fold less active than M1-IE2 RNA in cleaving substrate ie2-39, possibly because the P4 mutations abolished the catalytic activity of the ribozymes (*p* < 0.01).

Detailed gel-shift assays indicate that the binding affinities of V661-IE2-C and M1-IE2-C to substrate ie2-39, as measured by the dissociation constant (Kd), are similar to those of V661-IE2 and M1-IE2 (Table 1). Since V661-IE2-C and M1-IE2-C are non-functional but contain identical guide sequence complementary to ie2-39 and exhibit similar affinity to ie2-39 as V661-IE2 and M1-IE2, these ribozymes serve as controls for the antisense effect in our experiments.

### 2.2. Expression of Ribozymes in Human Cell Culture

The DNA sequences coding for V661-IE2, M1-IE2, V661-IE2-C, and M1-IE2-C were subcloned into LXSN retroviral vector and placed under the constitutively expressed U6 RNA promoter [19,21,38]. To construct cell lines that express M1GS RNAs, we generated retroviral vectors that contained the genes for M1GS RNAs by transfecting LXSN-M1GS DNAs into the amphotropic packaging cells (PA317). We subsequently infected human U373MG cells with the retroviruses and generated stable cell lines expressing the M1GSs. A cell line expressing ribozyme M1-TK, which targeted HSV-1 TK mRNA, was constructed to control for M1GS RNA with a mismatched guide sequence [22]. No cleavage of substrate ie2-39 by M1-TK was observed *in vitro* (data not shown, Table 1).

**Figure 2:**
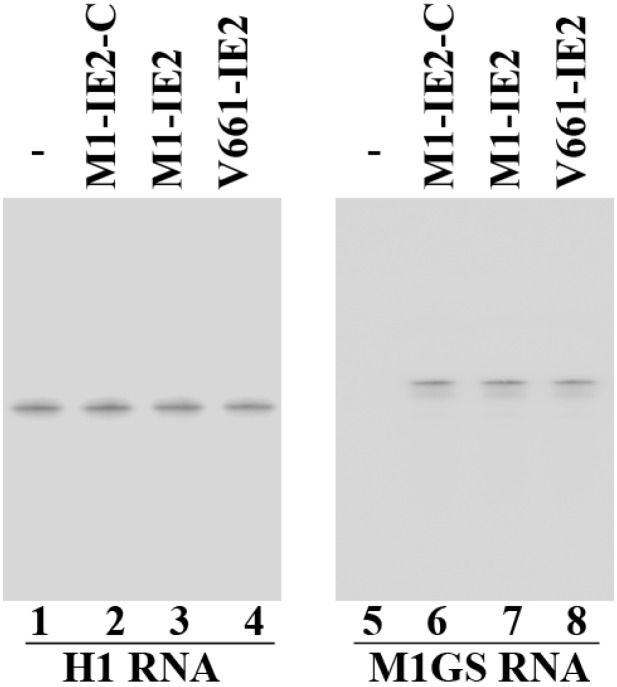
M1GS ribozymes expression with Northern blot analysis in parental U373MG cells (-, lanes 1 and 5) and cells with M1-IE2-C (lanes 2 and 6), M1-IE2 (lanes 3 and 7), and V661-IE2 (lanes 4 and 8). H1 RNA control (lanes 1–4).

Northern blot analysis was performed to assay M1GS RNA expression in individual cell clones (Figure 2, lanes 5–8). Human H1 RNA was used as the loading and internal control (Figure 2, lanes 1–4) [14,36]. No difference in cell growth and viability was observed for up to two months between cell lines expressing M1GS and a control line with empty vector LXSN DNA, suggesting that ribozymes did not result in significant cytotoxicity (data not shown). For subsequent experiments, only cell lines exhibiting similar ribozyme expression levels were used.

### 2.3. Enhanced Reduction of HCMV IE2 Expression in Cells Expressing the Engineered Ribozymes

Cells were infected with HCMV at a multiplicity of infection (MOI) of 1. Total RNAs were then isolated from these cells. HCMV immediate early 5kb RNA, which is expressed from the region spanning viral UL106 and UL111 with its 5' end at nucleotide position 159627 and its putative 3’ end at position 154829 of the HCMV (AD169) genome [1,39], was used as an internal loading control for IE2 mRNA expression.

**Figure 3:**
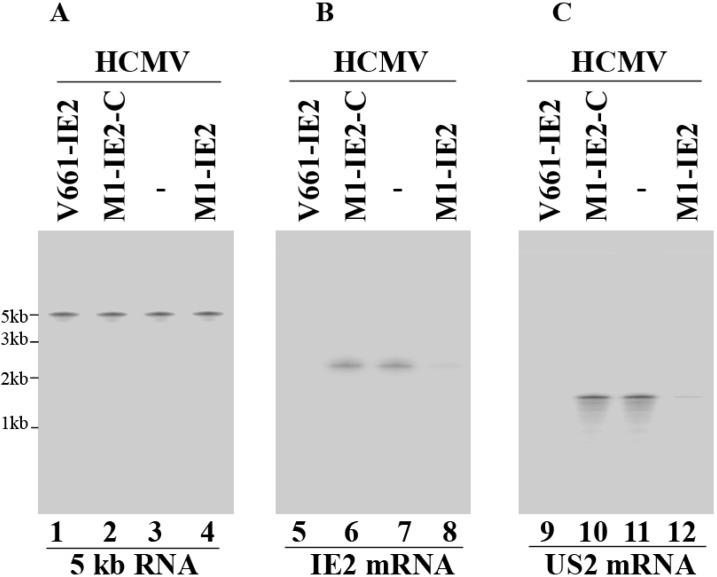
Northern analysis of the levels of human cytomegalovirus (HCMV) mRNAs. Cells (*n* = 1 × 10^6^) were infected with HCMV (MOI = 1) and were harvested at either 8 (**A**–**B**) or 24 h (**C**) post-infection. We used RNAs isolated from parental U373MG cells (-, lanes 3, 7, and 11) and cell lines that expressed V661-IE2 (lanes 1, 5, and 9), M1-IE2-C (lanes 2, 6, and 10), and M1-IE2 (lanes 4, 8, and 12). RNA samples were hybridized to a DNA probe coding for HCMV 5 kb RNA (lanes 1–4), IE2 mRNA (lanes 5–8), and US2 mRNA (lanes 9–12).

Figure 3 shows northern analysis of IE2 mRNA levels, which are quantitated in Table 2. A reduction of about 98% and 75% (*p* < 0.04) in IE2 mRNA expression was detected in cells that expressed V661-IE2 and M1-IE2, respectively (Figure 3, lanes 5 and 8).

In comparison, cells that expressed M1-IE2-C and V661-IE2-C showed little reduction (<10%) (Figure 3, lane 6, data not shown) (Table 2). An antisense effect could explain the low level of inhibition found in cells that expressed M1-IE2-C and V661-IE2-C. This is because these control ribozymes contained the same guide sequence as M1-IE2 and V661-IE2 but did not exhibit catalytic activity. These observations suggest that the significant reduction of IE2 mRNA expression in cells that expressed V661-IE2 and M1-IE2 was due to the catalytic cleavage of the target mRNA by these ribozymes. IE2 mRNA cleavage products were not detected in our northern analyses possibly due to degradation by intracellular RNases.

**Table 2:** HCMV mRNA and protein expression in cells expressing M1-TK, M1-IE2-C, V661-IE2-C, M1-IE2, or V661-IE2, and cells not expressing any ribozyme (U373MG). The values represent the levels of inhibition of gene expression as compared to the levels of inhibition in U373MG cells.

	Viral Gene Class	Ribozymes					
		U373MG	M1-IE2-C	V661-IE2-C	M1-IE2	V661-IE2	M1-TK
IE2 mRNA	α	0%	6%	5%	75% ± 7%	98% ± 7%	1%
US2 mRNA	β	0%	1%	1%	70% ± 6%	96% ± 8%	0%
IE2 protein	α	0%	4%	5%	75% ± 8%	98% ± 8%	0%
UL44 protein	β,γ	0%	1%	0%	71% ± 8%	95% ± 7%	1%
UL99 protein	γ	0%	0%	1%	72% ± 7%	94% ± 6%	0%
gH protein	γ	0%	0%	0%	71% ± 8%	98% ± 7%	1%

The values represent arithmetic means of three independent experiments performed in triplicate. *p* < 0.04. The values of standard deviation that were less than 5% are not shown.

**Figure 4:**
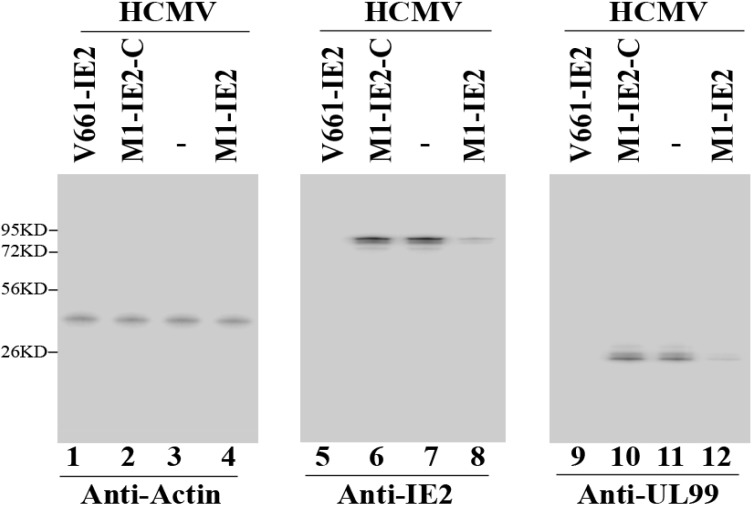
Levels of HCMV proteins observed by Western Blot. Actin control (lanes 1–4), IE2 (lanes 5–8), and UL99 (lanes 9–12). Cells (*n* = 1 × 10^6^) were infected with HCMV (MOI = 1) and were harvested at either 24 (lanes 1–8) or 72 h (lanes 9–12) post-infection.

IE2 protein levels in M1GS-expressing cells are expected to decrease due to the corresponding reduction in the level of IE2 mRNA. Proteins were isolated from cells, separated in gels, and transferred to membranes. The membranes were stained with an anti-IE2 antibody (anti-IE2) (Figure 4, lanes 5–8), and the expression levels of HCMV IE2 protein were determined. Actin was used as an internal and loading control (Figure 4, lanes 1–4). The results are summarized in Table 2 from three independent experiments: 98% and 75% reduction in IE2 protein expression (*p* < 0.04) was found in cells with V661-IE2 and M1-IE2 RNA, respectively. In comparison, little reduction (<10%) was detected in cells with V661-IE2-C, M1-IE2-C, and M1-TK RNAs (Table 2).

### 2.4. Increased Reduction of HCMV Infection by the Ribozymes

The expressions of HCMV β (early) and γ (late) genes are expected to be decreased due to the inhibition of IE2 expression [1,33]. To explore this, US2 mRNA level (a β mRNA) (Figure 3, lanes 9–12) as well as UL99 protein level (a γ protein) (Figure 4, lanes 9–12) were determined. We used HCMV 5 kb RNA and human actin as the internal loading controls. Our results demonstrated 95%–96% and 71%–73% reduction (*p* < 0.04) in the expression levels of these genes in cells with V661-IE2 and M1-IE2, respectively. Limited inhibition was found in cells with M1-IE2-C, V661-IE2-C, and M1-TK (Figure 3 and Figure 4, Table 2). Similar results were also observed in the expression of HCMV polymerase processivity factor UL44 (a β protein) and viral glycoprotein H (gH) (a γ protein) (Table 2). Thus, V661-IE2 and M1-IE2 expressing cells appeared to exhibit overall viral β and γ gene expression inhibition.

**Figure 5:**
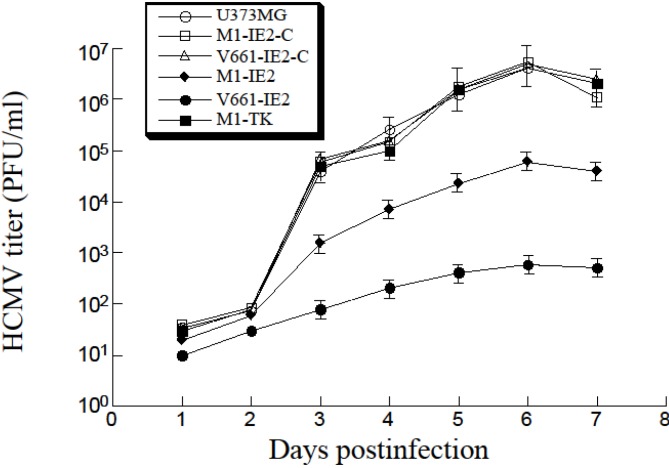
HCMV growth in various cell lines expressing M1GS and the parental U373MG cells. These values are the average from three experiments with error bars showing the standard deviation. *p* < 0.04.

The ribozymes also appeared to inhibit viral growth. After 5 days post-infection, we observed a 3500- and 100-fold reduction (*p* < 0.04) in viral titers in cells that expressed V661-IE2 and M1-IE2, respectively (Figure 5). In comparison, we observed no reduction in cells with control M1-IE2-C, V661-IE2-C, or M1-TK ribozymes (Figure 5, data not shown).

## 3. Discussion

Ribozyme-based gene targeting represents a promising therapeutic approach [5]. For ribozyme-mediated gene targeting to work efficiently, M1GS needs to be highly specific, catalytically efficient, and easily delivered to the target of choice. We have constructed M1GS RNAs that target the exon 5 region of HCMV IE2 mRNA and have shown that the ribozymes target the substrate efficiently *in vitro*. Moreover, 98% reduction in the expression level of IE2 and 3,500-fold reduction in viral growth were observed in cells with a ribozyme variant (*i.e.*, V661-IE2). In comparison, we detected little reduction (<10%) in IE2 expression and HCMV growth in cells with V661-IE2-C and M1-IE2-C. While non-functional, V661-IE2-C and M1-IE2-C contained identical guide sequence and showed similar binding affinity to the targeting mRNA as V661-IE2 and M1-IE2. Thus, our results imply that the overall observed inhibition of HCMV infection with M1-IE2 and V661-IE RNA was primarily due to specific ribozyme-mediated cleavage of the target mRNA.

Limited information is available about what limits M1GS-mediated gene-targeting efficacy in cultured cells [30]. Not much is known about how to improve the efficacy of M1GS RNA in blocking HCMV infection *in vivo*. In this report, M1GS was designed to target a DMS-accessible sequence of IE2 mRNA and was constitutively expressed by the U6 promoter. Our design should enhance the chance for M1GS to find the mRNA target. With the design, we postulated that the efficacy of RNase P ribozyme cleavage in cultured cells is dictated by its catalytic efficiency [(k_cat_/K_m_)^s^]. It is conceivable that enhancing catalytic activity of M1GS may reduce target mRNA expression more effectively *in vivo*. Indeed, V661-IE2, which was more efficient in cleaving ie2-39 *in vitro*, blocked IE2 expression and viral growth in cultured cells more effectively than M1-IE2, the wild type M1GS (Table 1 and Table 2). The difference between the *in vivo* efficacies of V661-IE2 and M1-IE2 (e.g., 98% *vs*. 75%) is less than the difference of *in vitro* cleavage efficiencies (more than 50-fold difference). A possible explanation is that about 1%–2% of the target mRNA substrates are unavailable for ribozyme cleavage because they may be bound by ribosome for translation prior to being bound by the ribozymes. Our work implies that increasing catalytic efficiencies [(k_cat_/K_m_)^s^] correlates with better ribozyme-mediated effect in reducing HCMV growth, and further suggests that improving the *in vitro* catalytic efficiencies of the ribozymes should lead to increased anti-HCMV efficacies in tissue culture. Thus, our study provides a guideline for the generation of highly effective RNase P ribozyme variants.

The antiviral effect of RNase P ribozymes appears to be specifically induced by the cleavage of the IE2 mRNA. First, cells expressing the ribozymes appeared normal with no toxicity for up to two months (data not shown). Second, the antiviral effect of the ribozyme (inhibition of viral growth) appears to be due to the decrease of IE2 expression. We detected overall reduction of viral β and γ gene expression (e.g., US2, UL44, UL99, and gH) in cells expressing functional M1-IE2 and V661-IE2 but not control M1-IE2-C or V661-IE2-C. The levels of reduction of IE2 expression correlate well with the levels of reduction in viral β and γ gene expression. In comparison, we detected no reduction in other viral immediate-early RNA (e.g., 5 kb RNA and UL36 mRNA) expression in these cells (Figure 3A, Table 2, data not shown) [22,40]. Thus, the antiviral effect of M1GS ribozyme may be induced by the cleavage of the IE2 mRNA.

Our study showed that variant V661-IE2 is more active in cleaving IE2 mRNA sequence than M1-IE2 *in vitro* (Table 1). Furthermore, a ribozyme targeting the HSV-1 TK mRNA sequence, V661-TK, was derived from variant V661. V661-TK also cleaved the TK mRNA sequence *in vitro* more efficiently than M1-TK, which was derived from the wild type ribozyme [31]. Thus, V661 may be used to construct highly efficient ribozymes to target any specific mRNAs. This variant has two point mutations: A_94_ → G_94_ and G_194_ → C_194_. Little is known about the roles of these two nucleotides in the M1GS-mediated cleavage of an mRNA substrate. Our study indicated that the point mutations at these two positions increased the overall cleavage rate (k_cat_/K_m_) without affecting the binding affinity (K_d_) to the mRNA substrate (Table 1). These results imply that the mutations may have no effect on the interactions of the ribozyme to the target mRNA. Perhaps these mutations increase the overall cleavage rate by stabilizing the active site and facilitating the folding of the overall structure of the ribozyme. Further biochemical characterization of V661 and other ribozyme variants will elucidate the mechanism by which mutations found in the variants increase the gene-targeting activity of the ribozymes and provide insight into the construction of highly effective ribozymes for gene targeting applications.

HCMV is a member of the human herpesvirus family [41,42]. Like other herpesviruses, HCMV can actively replicate or enter into latency [1]. When HCMV reactivates from latent infection and initiates lytic infection, IE2 is among the first proteins expressed and is essential for viral replication [1]. To study the functionality of M1GS ribozyme in HCMV latent infection, M1GS can be delivered into latently infected cells including CD34^+^ bone marrow progenitor cells. These studies will determine if RNase P ribozymes diminish IE2 expression and block HCMV reactivation. One potential challenge is to develop appropriate vectors for effective delivery and efficient expression of the ribozymes in CD34^+^ bone marrow progenitor cells. These and further studies should provide insight into the development of M1GSs as potentially effective anti-HCMV therapeutics.

## 4. Experimental Section

### 4.1. Viruses, Cells and Antibodies

HCMV (strain AD169) was grown in human astrocytoma U373MG cells and foreskin fibroblasts in Dulbecco’s modified Eagle medium (DMEM) [22,32]. The anti-UL44 and UL99 monoclonal antibodies were obtained from Virusys (Taneytown, MD, USA). Other antibodies were described previously [22,32].

### 4.2. Mapping of the Accessible Regions of HCMV IE2 mRNA in Cells

Detailed protocol for using dimethyl sulfate (DMS) to map the accessible regions of mRNA was described previously [36]. Briefly, DMS was incubated with HCMV-infected cells for 5–10 min. The cells were then lysed and the supernatant containing the cellular lysate was transferred to another tube for total RNA isolation with phenol-chloroform extraction and ethanol precipitation. Primer extension assays were performed with radiolabeled oligonucleotides in order to map the DMS modification sites [21,35], following the procedure described previously [36]. Phenol chloroform was used to extract the primer extension products followed by ethanol precipitation and separation in 8% denaturing gels. Sites that blocked primer extension, which represent potential locations modified by DMS, were identified in denaturing gels and analyzed using a STORM840 Phosphorimager [36].

### 4.3. Ribozyme Studies in Vitro

The ie2-39 DNA template was obtained by annealing oligonucleotide AF25 (5'-GGAATTCTAATACGACTCACTATAG-3') that contains a T7 promoter with oligonucleotide sie2-39 (5'-CGGGATCCTACTGGAATCGATACCGGCATGATTGACACCTATAGTGAGTCGTATTA-3'). Plasmid pFL117, pV661, and pC102, which encode M1 RNA, variant V661, and mutant C102, respectively, have been described previously [31,37]. C102 contains point mutations (A_347_C_348_ → C_347_U_348_, C_353_C_354_C_355_G_356_ → G_353_G_354_A_355_U_356_), which render it inactive [37]. Plasmids pFL117, pV661, and pC102 were used as templates to construct ribozymes M1-IE2, V661-IE2, and M1-IE2-C, respectively. Plasmid pV661-C, which was derived from pV661 and contained the mutations found in C102, was used as the template to construct ribozyme V661-IE2-C. The 5' PCR primer was AF25 while the 3' primer was M1IE23 (5'-CCCGCTCGAGAAAAAATGGTGCCGGTATCGATTCCAGTTGTGGAATTGTG-3'). Ribozymes and RNA substrate ie2-39 were synthesized with T7 RNA polymerase [12]. Kinetic analyses and gel shift assays were performed as described previously [31,43,44].

### 4.4. Construction of the M1GS-Expressing Cell Lines

The DNA sequences coding for the M1GSs were subcloned into retroviral vector LXSN and placed under the control of the U6 RNA promoter [12,21,45]. The retroviral vector DNAs containing the M1GS sequence were transfected into amphotropic PA317 cells. Culture supernatants containing retroviruses were used to infect human U373MG cells, and neomycin-resistant cells were selected in the presence of neomycin (600 µg/mL) (GE Healthcare, Piscataway, NJ) and were eventually cloned [12,21,45].

### 4.5. Assaying of Gene Expression and Viral Infection

Cells (*n* = 1 × 10^6^) were either mock-infected or infected with HCMV at a multiplicity of infection (MOI) of 1. Viral mRNA or protein samples were isolated from the infected cells at various time points as described previously [22,44]. Specifically, the samples used for the detection of the IE2 mRNA and protein expression were harvested at 8 and 24 h post-infection, respectively. Similarly, the samples used for the detection of the HCMV immediate-early UL36 mRNA were harvested at 8 h post-infection. The samples used for the detection of the US2 mRNA expression were harvested at 24 h post-infection while those used for the detection of the protein expression of UL44, UL99, and gH were harvested at 72 h post-infection. For controls, the samples for the detection of the 5kb RNA expression were harvested at 8 and 24 h post-infection while the samples for the detection of the actin protein expression were harvested at 24 and 72 h post-infection.

Western and northern blot analyses for the expression levels of HCMV proteins and mRNAs and ribozymes were performed as described previously [12,22,40]. In the northern blot analysis experiments, the RNA samples were separated in 1% agarose gels that contained formaldehyde, transferred to membranes, hybridized with the [^32^P]-radiolabeled DNA probes that contained the HCMV DNA sequences or the DNA sequences coding for M1 RNA and H1 RNA, and analyzed with a STORM840 Phosphorimager [12,22,40]. The radiolabeled DNA probes used in the northern blot analysis experiments were synthesized from plasmids using a random primed labeling kit (Roche Applied Science, Indianapolis, IN).

In the western blot analysis experiments, the protein samples were separated on 9% (v/v) SDS-polyacrylamide gels cross-linked with N,N-methylenebisacrylamide. The separated proteins were transferred electrically to nitrocellulose membranes and reacted with the antibodies against human actin and HCMV proteins. The membranes were subsequently stained with a chemiluminescent substrate with the aid of a Western chemiluminescent substrate kit (GE Healthcare) and quantitated with a STORM 840 Phosphorimager [12,22,40].

In order to accurately assay the viral mRNA/protein expression and increase the sensitivities of our detection assays, a series of diluted RNA and protein samples (1–100-fold dilution) were used and quantitation was performed in the linear range of RNA and protein detection. For example, two-fold changes in RNA and protein samples resulted in a two-fold change in signal bracketing the range of experimental values in our experiments. Furthermore, to obtain an accurate measurement of the expression level of a specific mRNA or protein, we carried out three separate and independent experiments. In each of these three experiments, cells were grown in triplicate and samples were collected from these cultures and analyzed. The results were the arithmetic average of the three experiments [12,22,40].

To study the M1GS-mediated inhibition of viral replication, U373MG cells (*n* = 5 × 10^5^) expressing M1GS were infected with HCMV at an MOI of 1. Viral stocks were prepared from cells harvested at 1 day interval for 7 days post-infection, and viral titers in the stocks were assayed by infecting human foreskin fibroblasts in triplicate, following previously described procedures [12,22].

### 4.6. Statistical Analysis

Statistical significance was determined using Student’s *t* test for paired samples [46]. All the experiments were carried out in triplicate, and repeated three times. We considered differences statistically significant at a value of *p* ≤ 0.05.

## 5. Conclusions

HCMV represents a significant global public health concern and developing novel anti-HCMV strategies is critical for the treatment and prevention of HCMV infection and associated diseases. RNase P ribozyme can act as a sequence-specific gene-targeting agent with promising applications in clinical settings. Here, we report the anti-HCMV activity of a ribozyme derived from a novel RNase P variant in cultured cells. The ribozyme variant cleaved the target IE2 mRNA *in vitro* and shut down IE2 expression in human cells more efficiently than the wild type ribozyme. Furthermore, cells expressing the variant exhibited more than a 3500-fold reduction in HCMV growth. Our study demonstrates that RNase P ribozyme variants are efficient in reducing HCMV infection and are potentially useful for anti-viral therapeutic applications.
